# Asymmetric Modulation of Brain Connectivity by Anodal Transcranial Direct Current Stimulation in Healthy Individuals: A Single‐Blind, Randomized Sham‐Controlled Trial

**DOI:** 10.1002/hbm.70218

**Published:** 2025-05-01

**Authors:** Tiffany Carther‐Krone, Zachary A. McAllister, Eun Hyung Choi, Lawrence Ryner, Ji Hyun Ko

**Affiliations:** ^1^ Department of Human Anatomy and Cell Science, Max Rady College of Medicine University of Manitoba Winnipeg Canada; ^2^ PrairieNeuro Research Centre Kleysen Institute for Advanced Medicine, Winnipeg Health Science Centre Winnipeg Canada; ^3^ Department of Radiology, Max Rady College of Medicine University of Manitoba Winnipeg Canada; ^4^ Graduate Program in Biomedical Engineering, Price Faculty of Engineering University of Manitoba Winnipeg Canada

**Keywords:** dorsolateral prefrontal cortex, fMRI, interference score, intrinsic connectivity, Stroop task, transcranial direct current stimulation

## Abstract

Transcranial direct current stimulation (tDCS) applied to the dorsolateral prefrontal cortex (DLPFC) has shown asymmetric behavioral effects, though the underlying neurophysiological mechanisms remain unclear. In this preliminary study with 34 healthy individuals, tDCS was applied to either the left or right DLPFC or a sham group. Behavioral and neurophysiological changes were examined by the Stroop test and resting‐state fMRI, respectively, which were measured before and after a 15‐min tDCS session. Seed‐to‐voxel connectivity analysis with seeds placed under the tDCS target regions (F3 and F4) showed no significant changes, but voxel‐to‐voxel whole‐brain intrinsic connectivity (IC) analysis revealed significant 3 × 2 interaction effects (stimulation site × time) in the right DLPFC (18 mm off from the F4). Post hoc analysis showed that only the right DLPFC stimulation led to an increase in IC from pre‐ to post‐stimulation. Consistent with this finding, right DLPFC stimulation improved Stroop task performance measured by increased interference score, which represents better inhibition of irrelevant information. These findings provide further insights into the hemispheric difference of tDCS effects and its underlying neurophysiological mechanisms. However, the small sample size limits the generalizability of the results and necessitates further research with a larger cohort for confirmation.

## Graphical abstract

Using transcranial direct current stimulation (tDCS) and fMRI, we compared the neurophysiological effects of anodal tDCS on the left vs. right dorsolateral prefrontal cortex (DLPFC). Our findings show that stimulation of the right (but not the left) DLPFC increases IC from pre‐ to post‐stimulation, demonstrating asymmetric brain connectivity.

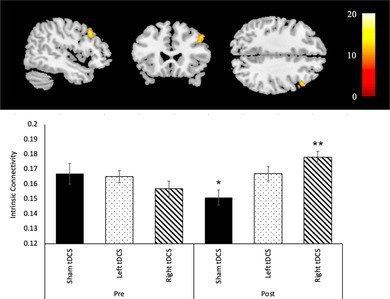

**Summary**

- Anodal stimulation of the right (but not the left) dorsolateral prefrontal cortex increases intrinsic connectivity from pre‐ to post‐stimulation, demonstrating asymmetric brain connectivity.

## 1 Introduction

Over the last 20 years, non‐invasive brain stimulation (NIBS) techniques, such as transcranial direct current stimulation (tDCS), have received increased interest (Ruffini et al. 2013). In tDCS, two electrodes are placed over the scalp to deliver a relatively weak current through the cortex, aiming to modulate brain function. This technique enables unique manipulation of neuronal excitability, allowing “perturbing and measuring” of brain activity and functions. As a painless, cost‐effective, and portable brain stimulation technology, tDCS produces long‐lasting effects when applied repeatedly and is often used to improve symptoms in individuals with brain disorders such as fibromyalgia, depression, and tinnitus (Lefaucheur et al. 2017). Both facilitatory and inhibitory effects of tDCS have been identified (Nitsche and Paulus 2000), typically depending on polarity, where anodal stimulation excites and cathodal stimulation inhibits the underlying membrane potential (Stagg and Nitsche 2011).

Early research on the neurophysiological mechanisms of tDCS focused on motor evoked potentials (MEPs) generated by transcranial magnetic stimulation (TMS) of the motor cortex (Nitsche and Paulus 2000; Priori et al. 1998). MEPs, typically measured using electromyography (EMG) by placing surface electrodes on muscles innervated by the motor cortex (e.g., the first dorsal interosseous muscle), are quantified based on their amplitude and latency to assess motor cortex excitability and motor pathway integrity. The motor cortex has been shown to be a symmetric region in terms of the effects of tDCS on MEPs, especially when hemispheric dominance and handedness are considered. For example, applying anodal tDCS to the left or right primary motor cortex (M1) increases MEP amplitudes in the corresponding muscle, while cathodal stimulation reduces them (Lang et al. 2004; Mordillo‐Mateos et al. 2012). Neuroimaging studies have suggested that the effects of M1 tDCS are not confined to the target regions but involve multiple brain regions (Lang et al. 2005; Polanía et al. 2011).

Beyond the motor cortex, the dorsolateral prefrontal cortex (DLPFC) has become a key area of interest due to its role in cognitive functioning. Unlike the M1, the neurophysiological measurements of tDCS effects are not readily available, and the prefrontal hemispheric specialization for different cognitive functions makes it more complicated to develop a model that can predict the effects of tDCS. Based on empirical evidence, it is now recommended that the left DLPFC be stimulated for depression while the right DLPFC be stimulated for addiction (Lefaucheur et al. 2017). However, our understanding of how tDCS modulates the respective behaviors and symptoms remains elusive. The brain‐behavior relationship can further be enlightened by using neuroimaging and surrogate neuropsychological tests (e.g., Stroop test (Stroop 1935)), the performance of which can immediately be modulated even after a single session of tDCS treatment.

Numerous studies have demonstrated that applying tDCS to the DLPFC enhances cognitive functions across various domains, including working memory (Andrews et al. 2011; Nissim et al. 2019; Simko et al. 2021; Stephens and Berryhill 2016), inhibitory control (Loftus et al. 2015; Metzuyanim‐Gorlick and Mashal 2016), and cognitive flexibility (Alizadehgoradel et al. 2021; Chrysikou et al. 2013). Neuroimaging research has revealed that the DLPFC is linked to several other brain regions, such as the thalamus, basal ganglia, orbitofrontal cortex, and various primary and secondary association areas in the neocortex, including posterior temporal, parietal, and occipital regions (Dosenbach et al. 2007; Tekin and Cummings 2002). Consequently, tDCS applied to the DLPFC can affect not only the target area but also the functionally connected regions. Indeed, previous studies have found that tDCS alters resting‐state functional connectivity networks, which are associated with cognitive improvement (Keeser et al. 2011; Krishnamurthy et al. 2015; Nissim et al. 2019; Polanía et al. 2011).

Despite growing evidence supporting tDCS‐induced changes in DLPFC functional connectivity, most studies using resting‐state fMRI data are guided by a priori hypotheses focused on a limited number of brain regions of interest (ROIs) or specific voxel locations (for examples, see Table S1). While this approach is valid and efficient, it may overlook significant areas influenced by tDCS that do not coincide with pre‐defined seed regions. Moreover, few studies have directly compared the effects of left versus right DLPFC stimulation on whole‐brain intrinsic connectivity. Given the hemispheric specialization of the DLPFC, understanding how tDCS differentially modulates connectivity patterns in each hemisphere is crucial for optimizing stimulation protocols for cognitive and clinical applications.

To address this issue, we conducted a tDCS‐fMRI experiment with healthy individuals to compare the neurophysiological effects of anodal tDCS on the left vs. right DLPFC. Resting‐state fMRI was used to measure functional connectivity before and after a 15‐min tDCS session. Participants also completed the Stroop test, in which the DLPFC has been consistently shown to be activated across studies (Chen et al. 2021b; Frings et al. 2018; MacDonald et al. 2000; Milham et al. 2003; Perrotta et al. 2021), to assess whether stimulation‐induced changes in connectivity corresponded to behavioral performance.

We hypothesized that anodal tDCS over the left versus the right DLPFC would modulate functional connectivity differently, reflecting the known functional asymmetry of the DLPFC. We also hypothesized that changes in functional connectivity would correlate with Stroop task performance, providing further evidence of the functional significance of tDCS‐induced alterations in connectivity.

## 2 Methods

### 2.1 Participants

In this randomized, single‐blind, sham‐controlled clinical trial, thirty‐nine participants were recruited from the community at large. Participant exclusion criteria were: (1) pregnant or breastfeeding women; (2) history of neurological or psychiatric disease; (3) metal implants or pacemaker; (4) abnormal MRI; (5) a Montreal Cognitive Assessment (MoCA) score lower than 25/30; (6) a Beck Depression Inventory II (BDI‐II) score of above 10; (7) severe hypertension; (8) cardiovascular disease; (9) family history of epilepsy. In total, 37 participants met the criteria and agreed to participate in the study. Of the 37 participants, three were excluded from the behavioral analysis due to incomplete Stroop data collection. This study was approved by the Biomedical Research Ethics Board of the University of Manitoba (HS19979 (H2016:074); clinicaltrials.gov/study/NCT03027869), and all participants provided written informed consent prior to participating.

### 2.2 Design and Measures

**FIGURE 1:**
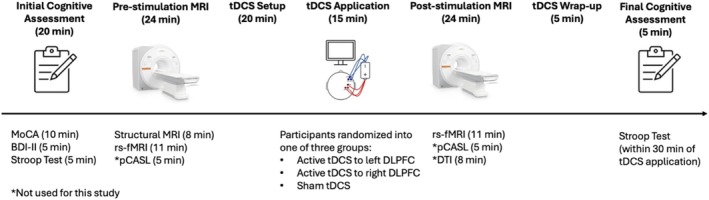
Overview of the experimental design depicting the procedure. Each session began with an initial cognitive assessment (MoCA, BDI‐II, Stroop task), followed by pre‐stimulation MRI. Participants then received 15 min of either anodal tDCS to the left or right DLPFC or sham tDCS based on the group to which they were randomly assigned. The session concluded with a post‐stimulation MRI and a final cognitive assessment (Stroop task).

All participants were cognitively and behaviorally assessed with the MoCA (Nasreddine et al. 2005), BDI‐II (Beck et al. 1996), and Stroop test (Stroop 1935). MoCA and BDI‐II administration occurred at the beginning of the session, and the Stroop test was given twice: once during the initial cognitive assessment and once after the tDCS session (within 30 min; Figure 1).

The MoCA detects cognitive impairments through tasks that assess cognitive domains such as attention and concentration, executive functions, memory, language, visual‐constructional skills, conceptual thinking, calculations, and orientation. A maximum of 30 points can be obtained, with a score of 26 or higher considered normal and a score of 25 or less indicative of mild cognitive impairment.

The BDI‐II is a 21‐item self‐administered Likert‐type depression scale with response options ranging from 0 to 3. Higher scores on the BDI‐II reflect more severe depressive symptomatology.

For the administration of the Stroop task, an automated neuropsychological assessment metrics program (Automated Neuropsychological Assessment Metrics 2016) was used. The Stroop task requires participants to press a computer key labeled as red, green, or blue to identify the colored stimulus presented on the screen. Participants were presented with three blocks, with each block lasting 45 s. In the first block (word reading), the words GREEN, RED, and BLUE were presented individually in white type on the screen, and the participant was instructed to press a corresponding key for each word (RED = 1, GREEN = 2, BLUE = 3). In the second block (color naming), a series of XXXXs was presented on the screen in either blue, red, or green type, and the participant was instructed to press the corresponding key based on color. In the third block (interference), individual words (RED, GREEN, BLUE) were presented in a color that did not match the name of the color depicted by the word, and the participant was instructed to press a corresponding computer key for the type color. Participants were instructed to respond as quickly and accurately as possible, aiming to complete as many trials as they could within the 45 s duration of each block. Stimulus presentation was user‐defined, meaning that a new stimulus only appeared after a response was made for the previous stimulus. Outcome measures included throughput (Thorne 2006), reported as the correct responses per minute of available response time, and interference score. Interference score was calculated using the method developed by Golden (1978), in which the interference score equals the number of correct responses for the third block minus predicted performance for the third block. Predicted performance of the third block is calculated by multiplying the number of correct responses for blocks one and two and dividing the result by the summation of correct responses for blocks one and two. Traditionally, the Stroop interference score is used to assess executive aspects of attentional control, such as freedom from distractibility, selective attention, response conflict, and response inhibition (MacLeod 1992; Melara and Algom 2003).

### 2.3 Application of tDCS and Functional Magnetic Resonance Imaging (fMRI) Acquisition

**FIGURE 2:**
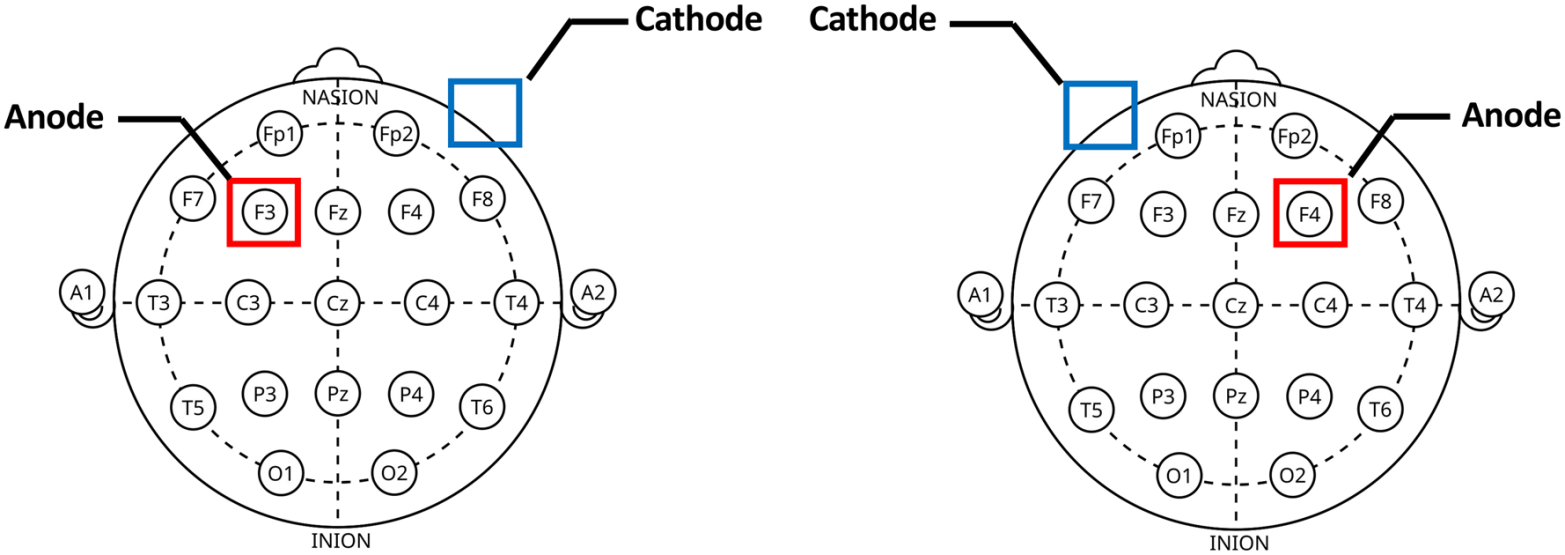
10–20 international EEG placement standard for tDCS targeting the left and right DLPFC, respectively.

After neuropsychological assessment, participants were assigned to one of three treatment groups using a random draw conducted by the researcher. The treatment groups included: application of tDCS to the left DLPFC (Anode placement of F3 for the 10–20 international EEG placement standard); application of tDCS to the right DLPFC (Anode placement of F4); and the sham stimulation (Figure 2). Stimulation was provided via a Soterix Medical 1 × 1 Transcranial Direct Current Low‐Intensity Stimulator with a 5 × 7 cm sponge at a current of 1.5 mA for 15 min. The ramp‐up and ramp‐down took 30 s each in the beginning and end of the 15‐min stimulation. For the sham condition, which was automatically controlled by “sham button” in the stimulator, initial ramp‐up was followed by immediate ramp‐down, and additional ramp‐up/down was provided in the last minute of the 15‐min stimulation period. Therefore, no current was administered for the middle 14 min.

The sponges and connected carbon‐rubber electrodes were placed on the participants’ head via an adjustable EASYstrap. For sham stimulation, the anode was placed on either the left (*n* = 8) or right (*n* = 5) DLPFC (F3 or F4). For all groups, the cathodal (reference) montage was placed on the contralateral supraorbital region. Previous research has demonstrated that anodal stimulation over the DLPFC with a different cathode placement (i.e., supraorbital region vs. opposite DLPFC placement) may induce different changes in DLPFC connectivity (Soleimani et al. 2021). For example, cathodal placement on the supraorbital region tends to have a more localized effect on the DLPFC, whereas placement on the contralateral DLPFC may induce stronger inter‐hemispheric connectivity changes (Soleimani et al. 2021). While these considerations are important, the use of a unilateral montage in the current study was driven by the specific aims of the study, which focused on modulating and assessing DLPFC connectivity in a more targeted and controlled manner. This approach reduces the potential for confounding inter‐hemispheric influences that could arise from using a bilateral montage.

All participants were blinded to their group allocation. Assessors and tDCS operators were also blind to the stimulation condition during the baseline assessment, as the random draw was performed immediately before the stimulator setup. The post‐stimulation assessment only included a computerized behavioral task.

All participants were scanned with 3 T MRI (Siemens/IMRIS Verio) equipped with a 12‐channel head coil located at the Kleysen Institute for Advanced Medicine at the University of Manitoba. During scanning, participants were instructed to keep their eyes open and not to fall asleep. Between the pre‐ and post‐tDCS fMRI scans, participants lay still in the scanner while tDCS was applied.

The structural T1 image was acquired using an MPRAGE sequence with TR/TE/TI = 2300/3.02/900 ms, 240 slices, flip angle = 9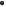, FOV = 256 mm x 256 mm, 1.0 mm^3^ (isotropic) resolution. After structural imaging, resting‐state fMRI scans were collected using the following parameters: repetition time [TR] = 2000 ms; echo time [TE] = 28 ms; flip angle = 77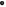 slice thickness = 4 mm; field of view [FOV] = 220 mm; voxel size = 3.4 X 3.4 X 4.0 mm; scan duration = 11 min. The same resting‐state fMRI was repeated after the 15‐min tDCS application.

### 2.4 Behavioral Analysis

Behavioral analysis was conducted using IBM version 27 SPSS software. One‐way analysis of variance (ANOVA) was used to compare the group (left, right, sham stimulation) differences in age, MoCA, and BDI‐II. Group differences in sex were analyzed using a chi‐square test. Group differences in Stroop interference scores were evaluated using a repeated‐measures ANOVA with group (left, right, sham stimulation) as the between‐subjects variable and time (pre, post) as the within‐subject variable. MoCA, which differed between groups, was included as a covariate. All analyses were considered significant at *p* < 0.05, using Bonferroni correction for multiple comparisons. Effect sizes were calculated using partial eta squared.

### 2.5 Functional Connectivity Analysis

MRI data processing was performed using the CONN toolbox (RRID:SCR_009550) version 22a (Nieto‐Castanon and Whitfield‐Gabrieli 2022). Standard preprocessing steps included co‐registration of functional and structural images, functional realignment and unwarp for subject motion estimation correction, slice‐timing correction, functional segmentation, structural segmentation and normalization, and smoothing.

**FIGURE 3:**
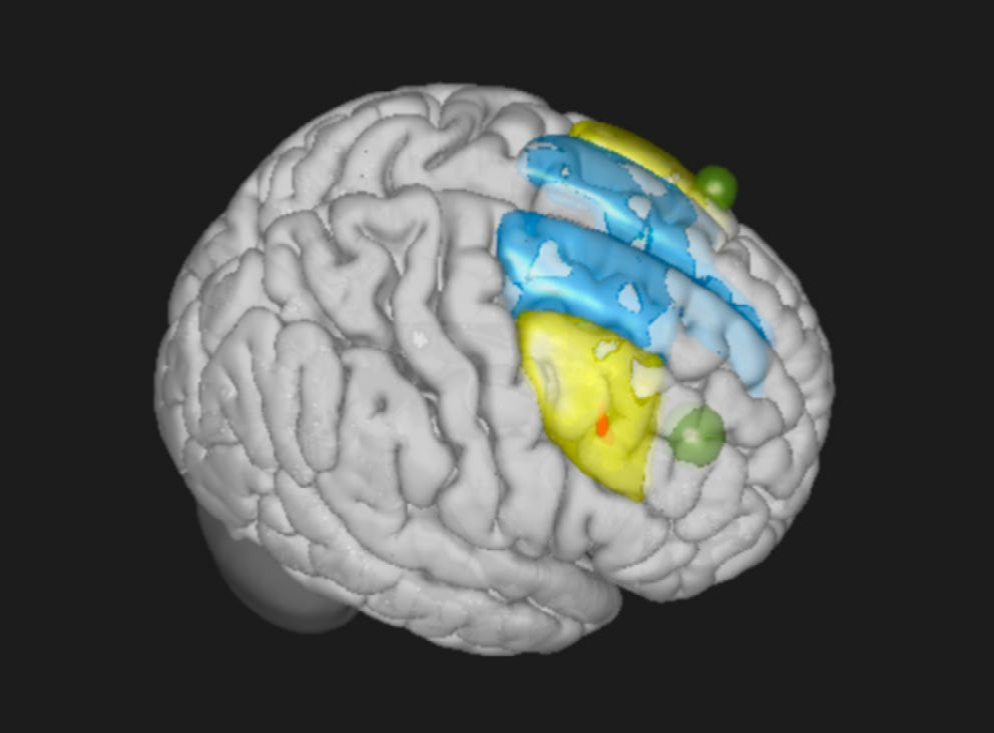
Pre‐defined seed regions included the bilateral superior frontal gyrus (blue) and mid frontal gyrus (yellow) from the CONN ROI atlas, as well as F3 and F4 (green), defined by an 8 mm radius sphere at MNI coordinates (*x* = −38, *y* = 34, *z* = 47) and (*x* = 39, *y* = 35, *z* = 48) respectively. Red cluster represents the voxels of significant interaction effects (time × stimulation sites) in intrinsic connectivity, which falls under the right middle frontal gyrus. Details are described in Figure 3, but it is visualized here to inform its relative location and size against the pre‐defined seed regions.

To investigate any changes in functional connectivity from the DLPFC, pre‐defined seed regions were used (Figure 3). Seed regions included the left and right superior frontal gyrus and mid frontal gyrus from the CONN ROI atlas, as well as F3 and F4, which were defined by creating 8 mm radius spheres at MNI coordinates (*x* = −38, *y* = 34, *z* = 47) and (*x* = 39, *y* = 35, *z* = 48) respectively (Scrivener and Reader 2022). Changes in functional connectivity were examined using a 3 (Group: left, right, sham stimulation) x 2 (Time: pre, post) ANOVA with the six pre‐defined ROIs as seed regions.

For the seed‐based connectivity analyses, a priori ROIs were placed in the MNI coordinates underneath the F3 and F4 based on group average MRI, which were determined by HDTargets software (Soterix Inc.). However, these strategies have limitations, which assume that the “hotspot” (the area of maximal electric field) of the tDCS effect would perfectly overlap with those a priori ROIs (Dmochowski et al. 2011). In other words, the brain region that has maximal electrical current may not necessarily colocalize with the one with maximal functional connectivity change. To address this issue, four additional anatomically defined ROIs were placed, i.e., the middle and superior frontal gyri. In addition, to account for the possibility that the maximal effects of tDCS fall outside of or do not sufficiently fit with those a priori ROIs, voxel‐to‐voxel intrinsic connectivity (IC) analysis was performed. The voxel‐to‐voxel IC measures the node centrality of each voxel and defines a voxel's centrality by the strength of its connectivity to the rest of the voxels in the whole brain. Mathematically, this is defined as the root mean square of correlation coefficients between a specific voxel and all other brain voxels. For the IC analysis, a 3 (Group: left, right, sham stimulation) x 2 (Time: pre, post) ANOVA was performed to determine any significant differences between groups.

For all analyses, the statistical threshold for a voxel to belong to a cluster was set to *p* < 0.001 (uncorrected) with a cluster extent threshold of *p* < 0.05 (false‐discovery‐rate corrected).

## 3 Results

### 3.1 Demographic Differences

Demographic data from all participants are summarized in Table 1. No significant differences in age, sex, or BDI scores were found between groups. However, the groups differed with respect to MoCA scores, *F* (2, 31) = 5.441, *p* = 0.009. Individuals in the sham group had ~1.4 points lower MoCA scores than those of the left (*p* = 0.033) and right (*p* = 0.018) stimulation groups. Nevertheless, all participants were above the cutoff for cognitive decline (≥ 26). No significant differences were found between left and right stimulation groups. The proportion of male to female participants did not differ in sham, left, or right stimulation groups. No differences in age, MoCA score, or BDI score were found between males and females in each group (Table S2).

**TABLE 1:** Demographic features.

	Sham	Left Stimulation	Right Stimulation	*p*
Subjects	12	10	12	
Age (Years)	45.67 ± 19.48	47 ± 17.5	50.5 ± 13.95	0.778
MoCA	27.33 ± 1.16	28.7 ± 1.06	28.75 ± 1.29	0.009
BDI	2.03 ± 2.57	3.3 ± 2.83	3.33 ± 3.39	0.516

*Note: p* values reflect ANOVA. Values are means ± SD.

### 3.2 Behavioral Results of the Stroop Task

**FIGURE 4:**
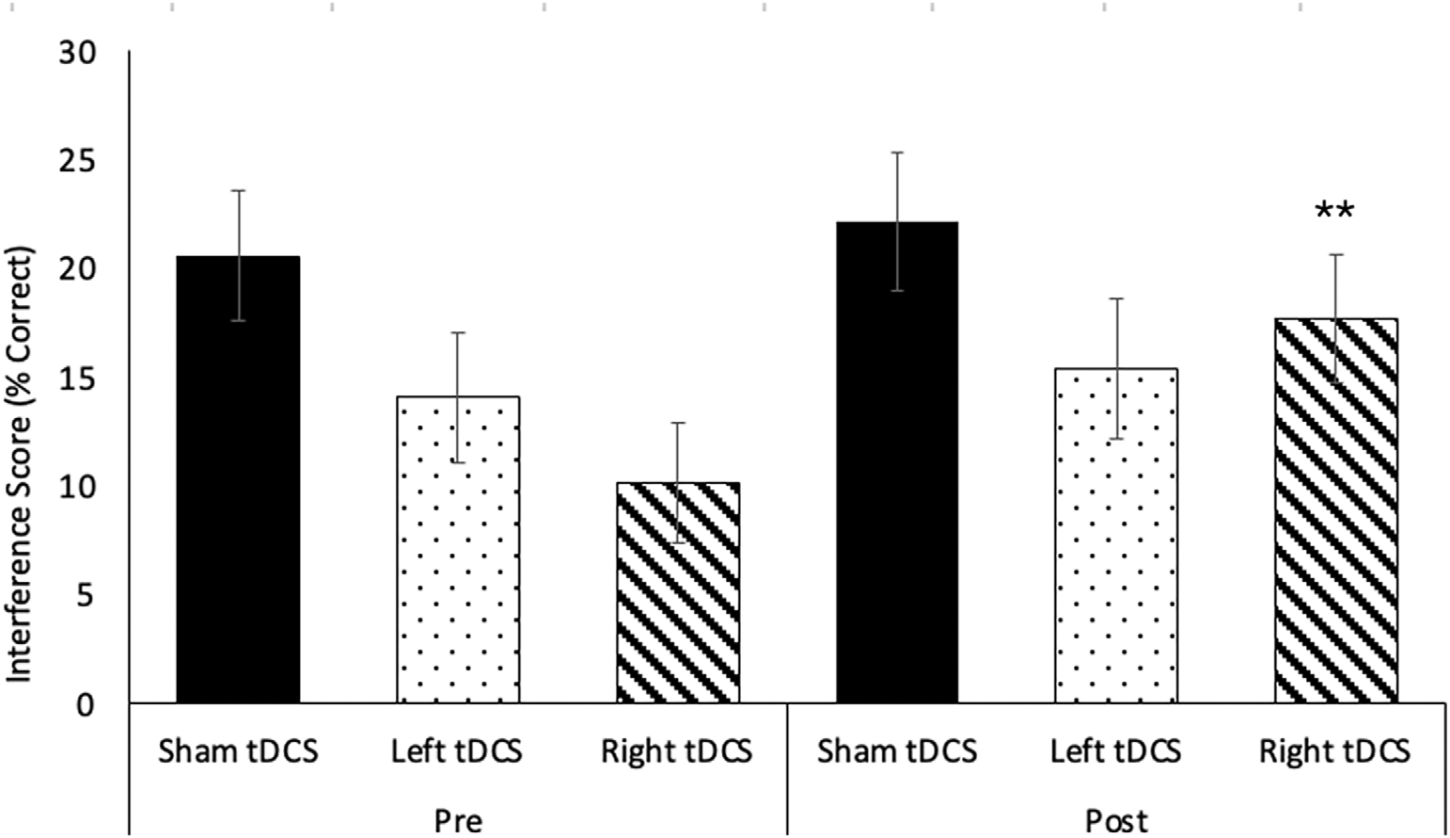
Changes in interference score of the Stroop test following tDCS. A 3 (treatment group: Left, right, sham tDCS) x 2 (time: Pre, post) repeated‐measures ANOVA revealed a significant interaction, *F* (2, 30) = 3.784, *p* = 0.034, *η*_p_^2^ = 0.201. **Post hoc tests showed a significant increase in interference score for the right tDCS group, *p* < 0.001. Values are means ± SE.

The primary behavioral outcome was the interference score (number of correct responses on block 3 minus the predicted color‐word score). On average, participants completed 53 trials per block (Median = 52.42; Range = 25.3–78.5). The number of trials completed did not differ between groups for blocks 1 to 3 in neither pre‐ nor post‐stimulation conditions, *p* > 0.3. No significant differences in interference score were noted across groups at baseline, *F* (2, 33) = 1.527, *p* = 0.233. A significant interaction was found, *F* (2, 30) = 3.784, *p* = 0.034, *η*_p_^2^ = 0.201. Post hoc tests showed a significant increase in interference score from pre‐ to post‐tDCS for the right tDCS group, *p* < 0.001 (Figure 4). No changes in interference score were found for the left tDCS or sham groups, *p* > 0.39.

### 3.3 Seed‐Based Connectivity Analysis

Seed‐to‐voxel functional connectivity analyses with a priori ROIs (i.e., the left and right superior frontal gyrus and mid frontal gyrus from the CONN ROI atlas, as well as the F3 and F4 at MNI coordinates: *x* = −38, *y* = 34, *z* = 47 and *x* = 39, *y* = 35, *z* = 48, respectively) did not reveal any significant clusters for an interaction effect (group × time, *p* > 0.1; Table S3).

### 3.4 Intrinsic Connectivity Analysis

**FIGURE 5:**
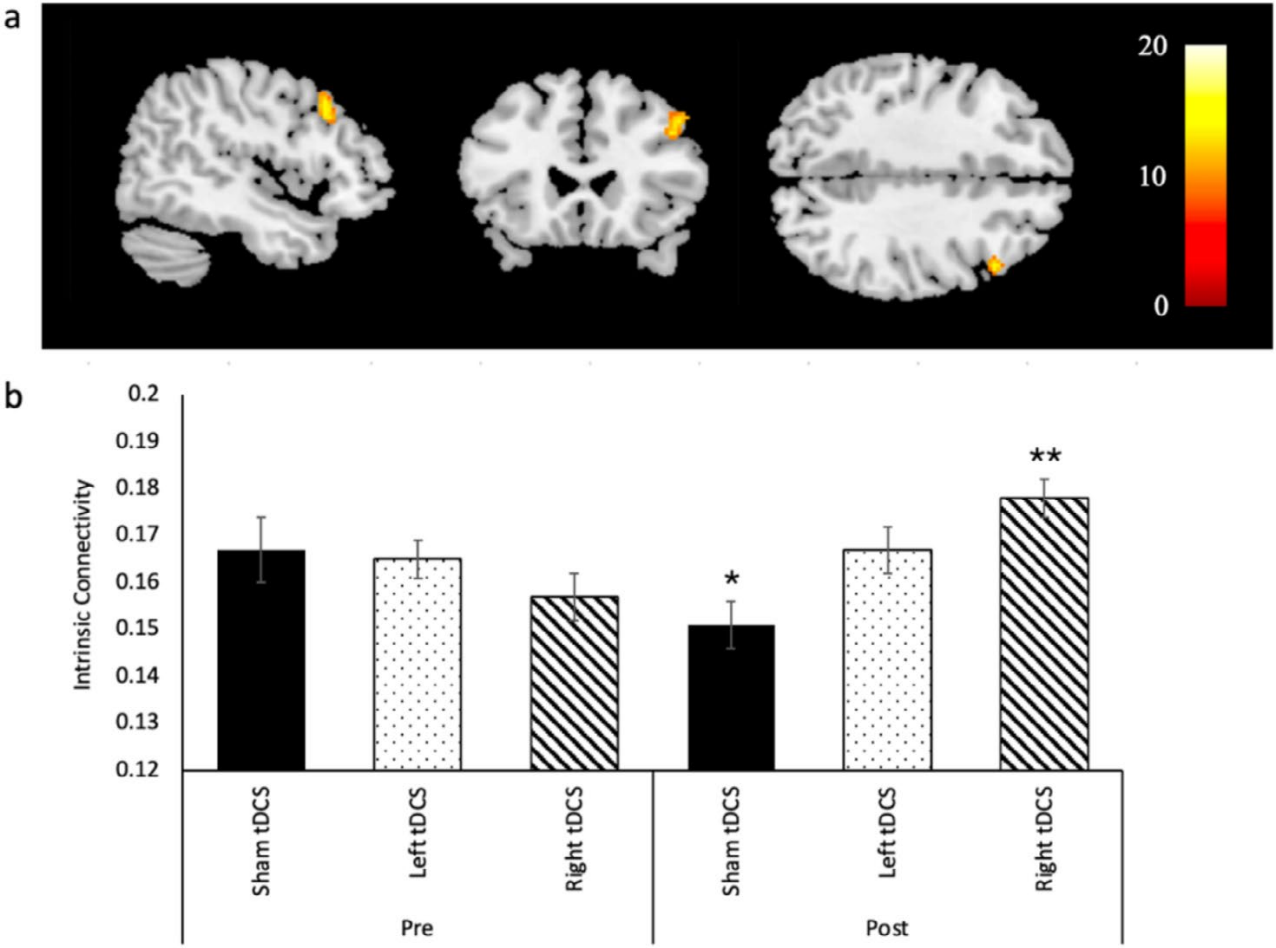
Changes in IC following tDCS (a) A voxel‐based 3 × 2 analysis for any difference between treatment groups (left, right, sham tDCS) and time (pre, post) revealed a significant cluster of IC changes in the right middle frontal gyrus (*p* = 0.04 cluster‐level FDR corrected, *p* < 0.001 peak‐level uncorrected, peak MNI coordinates +46, +22, +38). (b) Mean ICs were extracted from the identified cluster for post hoc analysis. No pre‐tDCS differences were found (*p* = 0.429). IC was decreased post‐tDCS compared to pre‐tDCS for the sham group (*p = 0.003), and it was increased for the right tDCS group (**p < 0.001). Values are means ± SE.

To locate the primarily affected brain regions by tDCS that may not be confined to the pre‐defined ROIs, voxel‐to‐voxel intrinsic connectivity analysis was performed. A 3 × 2 analysis for any difference between the three treatment groups (left, right, sham stimulation) and time (pre‐ and post‐tDCS) revealed a cluster located at the right middle frontal gyrus (*p* = 0.04 cluster‐level FDR corrected, *p* < 0.001 peak‐level uncorrected, *k* = 85, peak MNI coordinates +46, +22, +38; Figure 5a), which was 17.8 mm away from the ROI under the F4. Mean IC values were extracted from the cluster for post hoc analyses. No significant differences were found between groups in the pre‐tDCS condition, *F* (2, 31) = 0.870, *p* = 0.429. Post hoc tests also revealed a significant difference between pre‐ and post‐tDCS conditions for the sham and right tDCS groups, such that IC was decreased for the sham group, *p* = 0.003, and increased for the right tDCS group, *p* < 0.001 (Figure 5b).

## 4 Discussion

This study highlights the importance of the choice of analytic approach when investigating the effects of tDCS using fMRI. While relying on a priori ROIs for seed‐to‐voxel analysis resulted in negative findings, the voxel‐to‐voxel analysis accurately identified a significant cluster of the brain region that shows altered IC, which was 18 mm off from the peak underneath the F4 (the stimulation target; Figure 3). This finding that the significant cluster of functional connectivity change was not directly beneath the F4 electrode suggests that the effects of tDCS are not confined to the region of stimulation. Rather, these changes in connectivity reflect broader network modulations induced by stimulation, consistent with previous studies showing that tDCS modulates brain connectivity through a more distributed network, rather than being confined to the exact location of the electric field “hotspot” (Gomez‐Tames et al. 2020). Although the peak fell under the ROI of MFG, no significant results were observed for its seed‐to‐voxel analysis, potentially because the effects of tDCS were confined within the small cluster (85 vs. 22, 069 voxels within the resulting cluster vs. MFG‐ROI, respectively). Using the entire right MFG as a seed may not have been specific enough to elucidate the significant changes in functional connectivity induced by tDCS.

The voxel‐to‐voxel IC analysis discovered significant interaction effects that were confined only within the right DLPFC. Post hoc analysis revealed that this effect was predominantly driven by IC increases in the right DLPFC stimulation group (*p* < 0.001; cf. significant decrease of IC was also noted in the sham stimulation group, which is discussed separately below). Consistent with this finding, right DLPFC stimulation improved Stroop task performance, measured by increased interference score. These results highlight asymmetry in DLPFC function and how it responds to externally applied electrical stimulation, which may represent different neurophysiological properties. A possible explanation involves the asymmetric distribution of neurotransmitters for the left and right DLPFC. Post‐mortem studies have shown significant hemispherical differences in the distribution of neurotransmitters such as GABA and dopamine (Glick et al. 1982), though little research has examined this within the DLPFC. Prior studies have demonstrated that inhibitory repetitive transcranial magnetic stimulation (rTMS) to the left vs. right DLPFC differently affects task‐induced striatal dopamine release (Cho and Strafella 2009; Ko et al. 2008a), suggesting that these hemispheric differences may originate from the intrinsically asymmetric properties of the frontal lobes. Together with the present study, these findings suggest that caution is necessary when designing tDCS experiments targeting areas beyond the primary motor cortex where neuroexcitability changes are harder to measure.

Lesion studies highlighted that the superior medial prefrontal region is the most critical area for performing the Stroop task (Stuss and Alexander 2007), and neuroimaging studies (Aron et al. 2007; Chevrier et al. 2007; Li et al. 2006) have increasingly linked behavioral changes from the Stroop task to the DLPFC. However, there is less consensus regarding the lateralization of activity in this region. The conflict‐monitoring hypothesis (Botvinick et al. 1999, 2001) suggests that the anterior cingulate cortex (ACC) detects conflict and recruits the DLPFC for top‐down cognitive control (MacDonald et al. 2000). While correlates of top‐down control were originally thought to be left lateralized in the frontal cortex (Swick and Jovanovic 2002), more recent findings suggest lateralization is task‐dependent—the left DLPFC is involved in the expectation of conflicts, while the right DLPFC is more involved in processing the conflicts (Vanderhasselt et al. 2009).

We found that only the right DLPFC stimulation improved Stroop task performance as measured by increased interference score. This supports prior research indicating that the right DLPFC regulates cognitive control processes to reduce attentional conflict (Vanderhasselt et al. 2009), increasing error awareness (Sakai et al. 2013), and monitoring working memory (Ko et al. 2008b). This is consistent with previous research showing that anodal stimulation of the right DLPFC improves response inhibition (Chen et al. 2021b; Frings et al. 2018; Lapenta et al. 2014) while left DLPFC stimulation did not modulate Stroop interference (Baumert et al. 2020; Loftus et al. 2015). Examining the behavioral data alone, one may conclude that only the right DLPFC was causally related to Stroop interference effects, which highlights the functional hemispheric asymmetry of the DLPFC. However, the addition of neuroimaging findings suggests that the neurophysiological properties themselves were different between the left and the right DLPFC so that their responses to the tDCS also differed. In other words, it can also be speculated that the left DLPFC was more “resilient” to the externally injected electrical stimuli, and thus the behavior was not affected by the tDCS.

One unexpected finding was that the IC was decreased in the right DLPFC after a sham stimulation where the current was only applied for a 30 s ramp‐up and ramp‐down in the beginning and at the end of the 15‐min stimulation session. This finding raises the possibility that brief, low‐intensity stimulation contributed to the reduction in IC. However, when sham stimulation of the right DLPFC was compared to sham stimulation of the left DLPFC, there was no significant interaction effect between time (pre‐ vs. post‐) and electrode placement (left vs. right) (*F* (1, 11) = 0.559, *p* = 0.470). While the sample size of each of the left and right sham groups limits the interpretability of any observed effect (or lack thereof), these preliminary results suggest that the observed decrease in IC is not due to the brief ramp‐up and ramp‐down of stimulation itself. This suggests that other factors may explain these findings. One possibility may be participant expectation, as participants were told on the informed consent form that the purpose of the study was to “see what effects (good and bad) it may have on you and your cognitive function.” Although we only investigated the effects of tDCS in healthy individuals, a meta‐analysis suggests that healthy participants in research trials also experience placebo effects, albeit lesser degree compared to the patient population (Forsberg et al. 2017). Therefore, our participants would have likely perceived that their performance on the Stroop task was supposed to be influenced, and contributed to the findings. It is also possible that the observed decrease in IC is due to the sensitivity of the post hoc tests used to analyze brain connectivity. A computer simulation study has demonstrated that interaction effect tests with low sensitivity can increase the likelihood of false positives in control conditions (Ko 2024), which may explain the significant decrease if IC in the sham condition.

This study has several limitations. First, despite the considerable number of 34 participants, each group only had between 10 and 12 participants. The small sample size may limit the generalizability of the study because tDCS responses vary per individual. Second, behavioral results may be subject to practice effects. However, behavioral results within the sham group did not significantly differ, increasing the likelihood that results in the active stimulation groups were due to the stimulation itself. Another potential concern is the observed difference in MoCA scores between groups, with the sham group scoring lower than the stimulation groups. While all participants scored above the cutoff for cognitive impairment (≥ 26), we included MoCA scores as a covariate in all analyses to account for any potential influence on task performance or neural measures. We confirm that there was no significant interaction effect of MoCA as a covariate (*p* > 0.2).

Anodal stimulation is more commonly used for studies involving psychiatric disorders (Gianni et al. 2021), yet an increasing number of studies are experimenting with different cathode placements. While the current study used the supraorbital region as the reference site, future work should examine whether using a different reference site—such as the contralateral hemisphere—changes the observed intrinsic connectivity and behavioral effects. Future research should also explore the effects of cathodal stimulation. Since cathodal stimulation is generally associated with inhibitory effects on the cortex, which may suppress maladaptive plasticity (Chen et al. 2021a), looking at how cathodal stimulation influences intrinsic connectivity would provide a more comprehensive understanding of how tDCS modulates brain networks. Finally, while these findings demonstrate changes in intrinsic connectivity in response to DLPFC stimulation in healthy individuals, future work should examine how these effects translate to clinical populations.

In conclusion, this study underscores the importance of the choice of analytic approach when examining the effects of tDCS on brain connectivity and highlights the asymmetric neurophysiological properties of the DLPFC in response to tDCS stimulation. While anodal stimulation of the right DLPFC modulated intrinsic connectivity and improved Stroop task performance, these effects were not observed for left DLPFC stimulation, emphasizing the hemispheric laterality in executive function. These findings also show that tDCS‐induced connectivity changes extend beyond the immediate site of stimulation, reinforcing the notion that neuromodulation can influence broader brain networks.

## Conflicts of Interest

The authors declare no conflicts of interest.

## Supporting information


Data S1.

## Data Availability

The data that support the findings of this study are available on request from the corresponding author. The data are not publicly available due to privacy or ethical restrictions.

## References

[hbm70218-bib-0001] Alizadehgoradel, J. , V. Nejati , F. Sadeghi Movahed , et al. 2021. “Corrigendum to “Repeated Stimulation of the Dorsolateral‐Prefrontal Cortex Improves Executive Dysfunctions and Craving in Drug Addiction: A Randomized, Double‐Blind, Parallel‐Group Study” [Brain Stimul 13 (3) (2020) 582–593].” Brain Stimulation 14, no. 1: 182. 33418301 10.1016/j.brs.2020.12.012

[hbm70218-bib-0002] Andrews, S. C. , K. E. Hoy , P. G. Enticott , Z. J. Daskalakis , and P. B. Fitzgerald . 2011. “Improving Working Memory: The Effect of Combining Cognitive Activity and Anodal Transcranial Direct Current Stimulation to the Left Dorsolateral Prefrontal Cortex.” Brain Stimulation 4, no. 2: 84–89. 21511208 10.1016/j.brs.2010.06.004

[hbm70218-bib-0003] Aron, A. R. , T. E. Behrens , S. Smith , M. J. Frank , and R. A. Poldrack . 2007. “Triangulating a Cognitive Control Network Using Diffusion‐Weighted Magnetic Resonance Imaging (MRI) and Functional MRI.” Journal of Neuroscience 27, no. 14: 3743–3752. 10.1523/JNEUROSCI.0519-07.2007. 17409238 PMC6672420

[hbm70218-bib-0060] Automated Neuropsychological Assessment Metrics (Version 4) [Computer software]. 2016. Denver, CO: Vista LifeSciences, Inc.

[hbm70218-bib-0004] Baumert, A. , N. Buchholz , A. Zinkernagel , et al. 2020. “Causal Underpinnings of Working Memory and Stroop Interference Control: Testing the Effects of Anodal and Cathodal tDCS Over the Left DLPFC.” Cognitive, Affective, & Behavioral Neuroscience 20, no. 1: 34–48. 10.3758/s13415-019-00726-y PMC7012981 31183619

[hbm70218-bib-0005] Beck, A. T. , R. A. Steer , and G. K. Brown . 1996. BDI‐II, Beck Depression Inventory: Manual. Psychological Corp.

[hbm70218-bib-0007] Botvinick, M. M. , T. S. Braver , D. M. Barch , C. S. Carter , and J. D. Cohen . 2001. “Conflict Monitoring and Cognitive Control.” Psychological Review 108, no. 3: 624–652. 11488380 10.1037/0033-295x.108.3.624

[hbm70218-bib-0006] Botvinick, M. , L. E. Nystrom , K. Fissell , C. S. Carter , and J. D. Cohen . 1999. “Conflict Monitoring Versus Selection‐For‐Action in Anterior Cingulate Cortex.” Nature 402, no. 6758: 179–181. 10647008 10.1038/46035

[hbm70218-bib-0008] Chen, J. L. , A. Schipani , C. P. Schuch , et al. 2021a. “Does Cathodal vs. Sham Transcranial Direct Current Stimulation Over Contralesional Motor Cortex Enhance Upper Limb Motor Recovery Post‐Stroke? A Systematic Review and Meta‐Analysis.” Frontiers in Neurology 12: 626021. 10.3389/fneur.2021.626021. 33935936 PMC8083132

[hbm70218-bib-0009] Chen, T. , H. Wang , X. Wang , et al. 2021b. “Transcranial Direct Current Stimulation of the Right Dorsolateral Prefrontal Cortex Improves Response Inhibition.” International Journal of Psychophysiology 162: 34–39. 33497765 10.1016/j.ijpsycho.2021.01.014

[hbm70218-bib-0010] Chevrier, A. D. , M. D. Noseworthy , and R. Schachar . 2007. “Dissociation of Response Inhibition and Performance Monitoring in the Stop Signal Task Using Event‐Related fMRI.” Human Brain Mapping 28, no. 12: 1347–1358. 17274022 10.1002/hbm.20355 PMC6871417

[hbm70218-bib-0011] Cho, S. S. , and A. P. Strafella . 2009. “rTMS of the Left Dorsolateral Prefrontal Cortex Modulates Dopamine Release in the Ipsilateral Anterior Cingulate Cortex and Orbitofrontal Cortex.” PLoS One 4, no. 8: e6725. 19696930 10.1371/journal.pone.0006725 PMC2725302

[hbm70218-bib-0012] Chrysikou, E. G. , R. H. Hamilton , H. B. Coslett , A. Datta , M. Bikson , and S. L. Thompson‐Schill . 2013. “Noninvasive Transcranial Direct Current Stimulation Over the Left Prefrontal Cortex Facilitates Cognitive Flexibility in Tool Use.” Cognitive Neuroscience 4, no. 2: 81–89. 23894253 10.1080/17588928.2013.768221 PMC3719984

[hbm70218-bib-0013] Dmochowski, J. P. , A. Datta , M. Bikson , Y. Su , and L. C. Parra . 2011. “Optimized Multi‐Electrode Stimulation Increases Focality and Intensity at Target.” Journal of Neural Engineering 8, no. 4: 046011. 21659696 10.1088/1741-2560/8/4/046011

[hbm70218-bib-0014] Dosenbach, N. U. F. , D. A. Fair , F. M. Miezin , et al. 2007. “Distinct Brain Networks for Adaptive and Stable Task Control in Humans.” Proceedings of the National Academy of Sciences ‐ PNAS 104, no. 26: 11073–11078. 10.1073/pnas.0704320104 PMC1904171 17576922

[hbm70218-bib-0015] Forsberg, J. T. , M. Martinussen , and M. A. Flaten . 2017. “The Placebo Analgesic Effect in Healthy Individuals and Patients: A Meta‐Analysis.” Psychosomatic Medicine 79, no. 4: 388–394. 27922566 10.1097/PSY.0000000000000432

[hbm70218-bib-0016] Frings, C. , T. Brinkmann , M. A. Friehs , and T. van Lipzig . 2018. “Single Session tDCS Over the Left DLPFC Disrupts Interference Processing.” Brain and Cognition 120: 1–7. 29202318 10.1016/j.bandc.2017.11.005

[hbm70218-bib-0017] Gianni, E. , M. Bertoli , I. Simonelli , L. Paulon , F. Tecchio , and P. Pasqualetti . 2021. “tDCS Randomized Controlled Trials in No‐Structural Diseases: A Quantitative Review.” Scientific Reports 11, no. 1: 16311. 34381076 10.1038/s41598-021-95084-6 PMC8357949

[hbm70218-bib-0018] Glick, S. D. , D. A. Ross , and L. B. Hough . 1982. “Lateral Asymmetry of Neurotransmitters in Human Brain.” Brain Research 234, no. 1: 53–63. 6120746 10.1016/0006-8993(82)90472-3

[hbm70218-bib-0019] Golden, C. 1978. Stroop Color and Word Test. Stoelting Company.

[hbm70218-bib-0020] Gomez‐Tames, J. , A. Asai , and A. Hirata . 2020. “Significant Group‐Level Hotspots Found in Deep Brain Regions During Transcranial Direct Current Stimulation (tDCS): A Computational Analysis of Electric Fields.” Clinical Neurophysiology 131, no. 3: 755–765. 31839398 10.1016/j.clinph.2019.11.018

[hbm70218-bib-0022] Keeser, D. , T. Meindl , J. Bor , et al. 2011. “Prefrontal Transcranial Direct Current Stimulation Changes Connectivity of Resting‐State Networks During fMRI.” Journal of Neuroscience 31, no. 43: 15284–15293. 22031874 10.1523/JNEUROSCI.0542-11.2011 PMC6703525

[hbm70218-bib-0023] Ko, J. H. 2024. Interaction Effects Driven by Control Conditions: Does It Disqualify Your Results? Organization for Human Brain Mapping Annual Meeting.

[hbm70218-bib-0025] Ko, J. H. , O. Monchi , A. Ptito , M. Petrides , and A. P. Strafella . 2008b. “Repetitive Transcranial Magnetic Stimulation of Dorsolateral Prefrontal Cortex Affects Performance of the Wisconsin Card Sorting Task During Provision of Feedback.” International Journal of Biomedical Imaging 2008, no. 1: 143238. 18350118 10.1155/2008/143238 PMC2266810

[hbm70218-bib-0024] Ko, J. H. , O. Monchi , A. Ptito , P. Bloomfield , S. Houle , and A. P. Strafella . 2008a. “Theta Burst Stimulation‐Induced Inhibition of Dorsolateral Prefrontal Cortex Reveals Hemispheric Asymmetry in Striatal Dopamine Release During a Set‐Shifting Task ‐ a TMS‐[11C]Raclopride PET Study.” European Journal of Neuroscience 28, no. 10: 2147–2155. 19046396 10.1111/j.1460-9568.2008.06501.x PMC2967524

[hbm70218-bib-0026] Krishnamurthy, V. , K. Gopinath , G. S. Brown , and B. M. Hampstead . 2015. “Resting‐State fMRI Reveals Enhanced Functional Connectivity in Spatial Navigation Networks After Transcranial Direct Current Stimulation.” Neuroscience Letters 604: 80–85. 26240994 10.1016/j.neulet.2015.07.042

[hbm70218-bib-0028] Lang, N. , H. R. Siebner , N. S. Ward , et al. 2005. “How Does Transcranial DC Stimulation of the Primary Motor Cortex Alter Regional Neuronal Activity in the Human Brain?” European Journal of Neuroscience 22, no. 2: 495–504. 16045502 10.1111/j.1460-9568.2005.04233.x PMC3717512

[hbm70218-bib-0027] Lang, N. , M. A. Nitsche , W. Paulus , J. C. Rothwell , and R. N. Lemon . 2004. “Effects of Transcranial Direct Current Stimulation Over the Human Motor Cortex on Corticospinal and Transcallosal Excitability.” Experimental Brain Research 156, no. 4: 439–443. 14745467 10.1007/s00221-003-1800-2

[hbm70218-bib-0029] Lapenta, O. M. , K. D. Sierve , E. C. de Macedo , F. Fregni , and P. S. Boggio . 2014. “Transcranial Direct Current Stimulation Modulates ERP‐Indexed Inhibitory Control and Reduces Food Consumption.” Appetite 83: 42–48. 25128836 10.1016/j.appet.2014.08.005

[hbm70218-bib-0030] Lefaucheur, J.‐P. , A. Antal , S. S. Ayache , et al. 2017. “Evidence‐Based Guidelines on the Therapeutic Use of Transcranial Direct Current Stimulation (tDCS).” Clinical Neurophysiology 128, no. 1: 56–92. 27866120 10.1016/j.clinph.2016.10.087

[hbm70218-bib-0031] Li, C.‐s R. , C. Huang , R. T. Constable , and R. Sinha . 2006. “Imaging Response Inhibition in a Stop‐Signal Task: Neural Correlates Independent of Signal Monitoring and Post‐Response Processing.” Journal of Neuroscience 26, no. 1: 186–192. 16399686 10.1523/JNEUROSCI.3741-05.2006 PMC6674298

[hbm70218-bib-0032] Loftus, A. M. , O. Yalcin , F. D. Baughman , E. J. Vanman , and M. S. Hagger . 2015. “The Impact of Transcranial Direct Current Stimulation on Inhibitory Control in Young Adults.” Brain and Behavior: A Cognitive Neuroscience Perspective 5, no. 5: e00332‐n/a. 10.1002/brb3.332. PMC4389055 25874165

[hbm70218-bib-0033] MacDonald, A. W. , J. D. Cohen , V. A. Stenger , and C. S. Carter . 2000. “Dissociating the Role of the Dorsolateral Prefrontal and Anterior Cingulate Cortex in Cognitive Control.” Science 288, no. 5472: 1835–1838. 10846167 10.1126/science.288.5472.1835

[hbm70218-bib-0034] MacLeod, C. M. 1992. “The Stroop Task: The ‘Gold Standard’ of Attentional Measures.” Journal of Experimental Psychology. General 121, no. 1: 12–14.

[hbm70218-bib-0035] Melara, R. D. , and D. Algom . 2003. “Driven by Information: A Tectonic Theory of Stroop Effects.” Psychological Review 110, no. 3: 422–471. 12885110 10.1037/0033-295x.110.3.422

[hbm70218-bib-0036] Metzuyanim‐Gorlick, S. , and N. Mashal . 2016. “The Effects of Transcranial Direct Current Stimulation Over the Dorsolateral Prefrontal Cortex on Cognitive Inhibition.” Experimental Brain Research 234, no. 6: 1537–1544. 26821316 10.1007/s00221-016-4560-5

[hbm70218-bib-0037] Milham, M. P. , M. T. Banich , and V. Barad . 2003. “Competition for Priority in Processing Increases Prefrontal Cortex's Involvement in Top‐Down Control: An Event‐Related fMRI Study of the Stroop Task.” Cognitive Brain Research 17, no. 2: 212–222. 12880892 10.1016/s0926-6410(03)00108-3

[hbm70218-bib-0038] Mordillo‐Mateos, L. , L. Turpin‐Fenoll , J. Millán‐Pascual , et al. 2012. “Effects of Simultaneous Bilateral tDCS of the Human Motor Cortex.” Brain Stimulation 5, no. 3: 214–222. 21782545 10.1016/j.brs.2011.05.001

[hbm70218-bib-0039] Nasreddine, Z. S. , N. A. Phillips , V. Bédirian , et al. 2005. “The Montreal Cognitive Assessment, MoCA: A Brief Screening Tool for Mild Cognitive Impairment.” Journal of the American Geriatrics Society 53, no. 4: 695–699. 15817019 10.1111/j.1532-5415.2005.53221.x

[hbm70218-bib-0040] Nieto‐Castanon, A. , and S. Whitfield‐Gabrieli . 2022. “CONN Functional Connectivity Toolbox: RRID SCR_009550.” 10.1089/brain.2012.0073 22642651

[hbm70218-bib-0041] Nissim, N. R. , A. O'Shea , A. Indahlastari , et al. 2019. “Effects of In‐Scanner Bilateral Frontal tDCS on Functional Connectivity of the Working Memory Network in Older Adults.” Frontiers in Aging Neuroscience 11, no. 51: 51. 10.3389/fnagi.2019.00051. 30930766 PMC6428720

[hbm70218-bib-0042] Nitsche, M. A. , and W. Paulus . 2000. “Excitability Changes Induced in the Human Motor Cortex by Weak Transcranial Direct Current Stimulation.” Journal of Physiology 527, no. 3: 633–639. 10990547 10.1111/j.1469-7793.2000.t01-1-00633.x PMC2270099

[hbm70218-bib-0043] Perrotta, D. , V. Bianco , M. Berchicci , F. Quinzi , and R. L. Perri . 2021. “Anodal tDCS Over the Dorsolateral Prefrontal Cortex Reduces Stroop Errors. A Comparison of Different Tasks and Designs.” Behavioural Brain Research 405: 113215. 33662440 10.1016/j.bbr.2021.113215

[hbm70218-bib-0044] Polanía, R. , M. A. Nitsche , and W. Paulus . 2011. “Modulating Functional Connectivity Patterns and Topological Functional Organization of the Human Brain With Transcranial Direct Current Stimulation.” Human Brain Mapping 32, no. 8: 1236–1249. 20607750 10.1002/hbm.21104 PMC6870160

[hbm70218-bib-0045] Priori, A. , A. Berardelli , S. Rona , N. Accornero , and M. Manfredi . 1998. “Polarization of the Human Motor Cortex Through the Scalp.” Neuroreport 9, no. 10: 2257–2260. 9694210 10.1097/00001756-199807130-00020

[hbm70218-bib-0046] Ruffini, G. , F. Wendling , I. Merlet , et al. 2013. “Transcranial Current Brain Stimulation (tCS): Models and Technologies.” IEEE Transactions on Neural Systems and Rehabilitation Engineering 21, no. 3: 333–345. 22949089 10.1109/TNSRE.2012.2200046

[hbm70218-bib-0047] Sakai, H. , Y. Uchiyama , D. Shin , M. J. Hayashi , and N. Sadato . 2013. “Neural Activity Changes Associated With Impulsive Responding in the Sustained Attention to Response Task.” PLoS One 8, no. 6: e67391‐e. 23825657 10.1371/journal.pone.0067391 PMC3692459

[hbm70218-bib-0048] Scrivener, C. L. , and A. T. Reader . 2022. “Variability of EEG Electrode Positions and Their Underlying Brain Regions: Visualizing Gel Artifacts From a Simultaneous EEG‐fMRI Dataset.” Brain and Behavior: A Cognitive Neuroscience Perspective 12, no. 2: e2476. 10.1002/brb3.2476 PMC8865144 35040596

[hbm70218-bib-0049] Simko, P. , M. Pupikova , M. Gajdos , and I. Rektorova . 2021. “Cognitive Aftereffects of Acute tDCS Coupled With Cognitive Training: An fMRI Study in Healthy Seniors.” Neural Plasticity 2021: 6664479. 33953741 10.1155/2021/6664479 PMC8057875

[hbm70218-bib-0050] Soleimani, G. , M. Saviz , M. Bikson , et al. 2021. “Group and Individual Level Variations Between Symmetric and Asymmetric DLPFC Montages for tDCS Over Large Scale Brain Network Nodes.” Scientific Reports 11, no. 1: 1271. 10.1038/s41598-020-80279-0. 33446802 PMC7809198

[hbm70218-bib-0051] Stagg, C. J. , and M. A. Nitsche . 2011. “Physiological Basis of Transcranial Direct Current Stimulation.” Neuroscientist 17, no. 1: 37–53. 21343407 10.1177/1073858410386614

[hbm70218-bib-0052] Stephens, J. A. , and M. E. Berryhill . 2016. “Older Adults Improve on Everyday Tasks After Working Memory Training and Neurostimulation.” Brain Stimulation 9, no. 4: 553–559. 27178247 10.1016/j.brs.2016.04.001 PMC4957521

[hbm70218-bib-0053] Stroop, J. R. 1935. “Studies of Interference in Serial Verbal Reactions.” Journal of Experimental Psychology 18, no. 6: 643–662.

[hbm70218-bib-0054] Stuss, D. T. , and M. P. Alexander . 2007. “Is There a Dysexecutive Syndrome?” Philosophical Transactions of the Royal Society of London. Series B, Biological Sciences 362, no. 1481: 901–915. 17412679 10.1098/rstb.2007.2096 PMC2430005

[hbm70218-bib-0055] Swick, D. , and J. Jovanovic . 2002. “Anterior Cingulate Cortex and the Stroop Task: Neuropsychological Evidence for Topographic Specificity.” Neuropsychologia 40, no. 8: 1240–1253. 11931927 10.1016/s0028-3932(01)00226-3

[hbm70218-bib-0056] Tekin, S. , and J. L. Cummings . 2002. “Frontal–Subcortical Neuronal Circuits and Clinical Neuropsychiatry: An Update.” Journal of Psychosomatic Research 53, no. 2: 647–654. 12169339 10.1016/s0022-3999(02)00428-2

[hbm70218-bib-0057] Thorne, D. R. 2006. “Throughput: A Simple Performance Index With Desirable Characteristics.” Behavior Research Methods 38, no. 4: 569–573. 10.3758/bf03193886. 17393825

[hbm70218-bib-0058] Vanderhasselt, M.‐A. , R. De Raedt , and C. Baeken . 2009. “Dorsolateral Prefrontal Cortex and Stroop Performance: Tackling the Lateralization.” Psychonomic Bulletin & Review 16: 609–612. 19451392 10.3758/PBR.16.3.609

