# Supplementary material for: Asymmetric Modulation of Brain Connectivity by Anodal Transcranial Direct Current Stimulation in Healthy Individuals: A Single‐Blind, Randomized Sham‐Controlled Trial
Source: Hum Brain Mapp. 2025 May 1;46(7):e70218. doi: 10.1002/hbm.70218 (PMC12045925; doi:10.1002/hbm.70218)
Supplement: Supplementary file 1 — Data S1. [file HBM-46-e70218-s001.docx]

**Supplementary Materials**

**Supplementary Table S1.** **Qualitative summary of the demographic and experimental details of reviewed studies.**

*Please note that this is not meant to be a systemic review but was provided to qualitatively inform about overall trend of tDCS setup.

**Supplementary Table S2. Demographic features by sex.**

^a^For subjects, p-values reflect the Pearson X^2^ test; for age, MoCA and BDI, p-values reflect t-tests between males and females for each group. ^b^Values are means ± SD.

**Supplementary Table S3. Seed-based connectivity null effects**

| **Seed Region** | **Cluster (x,y,z)** | **Cluster Size** | **p-FDR** |
| --- | --- | --- | --- |
| Left SFG | +20 -74 -6 | 95 | 0.106633 |
| Right SFG | +44 -72 -2 | 28 | 0.770964 |
| Left MFG | +30 -32 +22 | 80 | 0.123733 |
| Right MFG | -44 -78 -42 | 45 | 0.455384 |
| Left DLPFC | +40 -32 +24 | 73 | 0.253622 |
| Right DLPFC | +4 10 -22 | 23 | 0.712476 |

To assess the null effects in the seed-based connectivity analysis, an additional analysis was carried out using a p-uncorrected threshold of .001 and a cluster-extent threshold of 1. For the first occurring cluster in each of the seed regions used in the seed-based connectivity analysis this table shows the cluster location, size and FDR corrected p-value.
